# The Cost of Nursing

**Published:** 1907-04-20

**Authors:** E. W. Morris

**Affiliations:** Secretary. London Hospital, Whitechapel, E.


### CORRESPONDENCE.

### The Cost of Nursing.

In an article in your issue of April 6th reference is made to the cost of nursing at the various hospitals, and the London Hospital is referred to as being the most expensive in this respect.

It is stated—quoting from Sir Henry Burdett's latest edition of "Hospitals and Charities"—that each occupied bed costs £12 7s. a year in nurses' salaries. This figure is obtained by dividing our nurses' total salaries—namely, £9,377—by 741, the number of beds occupied. It is not a very important point, but is perhaps worth mentioning that this sum of £9,377 includes the salaries of about 50 nurses who have no bedside work at all. All the hospitals perhaps suffer from a similar disadvantage, as the "cost per bed" in nursing has been arrived at in the same way in every case; but the London suffers more in that its non-bedside work is greater as compared with its bedside work than most, if not all, the other hospitals.

In the article referred to attention is drawn to the very great difference in the "cost per bed" for nursing at the various hospitals, and it is stated that as salaries paid to the individual nurses do not show this great variation, the difference in cost would appear to lie in the numbers. This is certainly true, but it is advisable that the public who support hospitals should know why there is such variation in the numbers. For instance, it is stated in Sir Henry's Annual that at the London there is one nurse to every 1.9 patients. This figure is arrived at by dividing 741, the number of beds, by 389, the number of nurses actually engaged at bedside work. The statement may be misleading.

Suppose, for instance, that at a particular hospital at a certain time there are 300 beds and 100 nurses, and that a year or two after there are still 300 beds but 150 nurses; it might be inferred that the figures simply show that the Board had decided to supply a nurse to every *two* patients instead of a nurse to *three* patients, as hitherto. The figures do not mean this, however. What they show, if figures can show anything, is that the Board considers that the nurse should work shorter hours, and in this hypothetical case two hours for every three she previously worked; not that she should attend fewer patients. Obviously, the shortening of the nurses' hours and lengthening of their holidays must increase the number of nurses employed at an institution.

Here every nurse gets three hours off duty, either in the morning or the afternoon—*i.e.*, by daylight—the whole day off duty once a fortnight, and a whole month's holiday in the year. And this is why the London employs 389 nurses to 741 beds; certainly not because we think a nurse can only manage 1.9 patients when on duty.

Yours faithfully, E. W. Morris,
Secretary.

London Hospital, Whitechapel, E.
April 10th, 1907.

. ·. As a basis of comparison between hospitals, the division of occupied beds among the ward staff works out very fairly. All hospitals are compelled to keep an extra staff for special cases, leave and off duty times, and it is precisely in the management of this extra staff that economy tells. The fact that day and night staffs are classed together in this mode of reckoning shows conclusively that the average number of beds to each nurse does not stand for the number which it is found she "can manage when on duty." To include the salaries of those engaged outside the walls in the average cost of nursing per bed may tend slightly to raise the average cost for hospitals with large out-patient departments, as compared with some small provincial hospitals. But as Mr. Morris points out, this is "not a very important point." In the first place, for purposes of profitable comparison, classification is essential. In the second place, the difference which the inclusion of the extra ward staff makes in the average is, in most instances, negligible. The London Hospital, for instance, when the salaries of the 50 nurses who "have no bedside work" is deducted, shows an average of £11 4s. per occupied bed for nursing, and is still higher for this item than other general hospitals, except St. George's, which shows an average for the total nursing staff of £12 2s.—Ed. The Hospital.

